# Medical Education

**Published:** 1858-07

**Authors:** J. H. Brown

**Affiliations:** Lawrenceburgh, Indiana


ARTICLE V.

### MEDICAL EDUCATION.

BY J. H. BROWN, M. D., LAWRENCEBURGH, INDIANA.

The subject of your circular has for many years, indeed, from the commencement of my professional life, engaged my earnest attention. I have watched, with growing interest, the movement abroad in the profession, looking to the consummation, so devoutly to be wished, when medical education should be placed, at least, in some degree, on a basis approaching the systems of general intellectual instruction now universally adopted, and in comparison with which, the hasty and superficial course of study now sanctioned by all our medical schools is absurd, unreasonable and suicidal to the honor and dignity of the medical profession; and if the time is ever to arrive, when, as of old, the *Doctor* (heaven save the mark!) is to become, as his name legitimately implies, the *doctus vir*, it must be by just such plain, outspoken efforts as (I am glad to see) you and your confreres are now making. I once had occasion, upon a festive reunion of professional brethren, to combat and refute the bold assertion, that the present profession were "si non doctiores, meliore imbuti doctrina:" a grosser absurdity was never uttered, and could only be excused on the ground, that, by the present authorized system of medical instruction, young men were everywhere admitted to the honors (?) of the profession, who could not possibly by it be learned, and scarcely technical. The legitimate practical result, to the profession, as such, leaving out of the question the sad results of incompetency and assumption, has been a gradual and steady decadence in the general estimation of the profession, and an increasing distrust among enlightened men, as to the certainty and truthfulness of the science. The insane desire to multiply medical schools all over the land, the "*cacoethes docendi*," which has so sorely smitten many of our brethren, who had, as Cowper says, skulls "that would not learn and could not teach," and the low estimate of professional excellence among ourselves, have done much to bring about this result; and we seem now to have reached the point, when, either a more rigid and common sense mode of preparation must be carried out, or we must be content to yield the ground to empiricism and imposture.

Upon a careful examination of the plan you propose, I am constrained to give it my hearty approval; and I do not know that I can add anything to its details. The separation of the elementary from the practical department of a course of lectures, is a vital point, indeed, the true point at issue. I have never been a public teacher, but you and your associates must have felt the absurdity of the position, when, in a short four months' course, you were compelled, by the exigencies of your requirements, to compress into your course the instruction suited to several grades of advancement, and by your vain efforts to *go over*, as the phrase is, all the topics necessary to be embraced in your course, what, in truth, is it but a "rudis, indigestaque moles?" The advanced student is restive, listless and impatient, under the infliction of what he has already mastered, as first principles; while, to the neophyte, practical and ultimate teaching is about as profitable and sensible as to clothe an infant with the toga virilis. To tell you the honest truth, my dear Doctor, I do not envy you your position, and one great reason why I have not aspired to so honorable a station has been, that I do not believe any man can do himself justice and acquit his conscience, in the present senseless arrangement of professorial requirement.

I am of opinion that the proposed plan, if properly carried out, would not only, as you say, not *necessarily* increase the number of professors required, but would in reality require a less number to accomplish a much greater result, and thus lessen, instead of increasing the expenses of the student, although this reason, *politic* as it may be, ought not to have an undue weight. I would not increase the temptations to young men to crowd our college halls, by holding out the idea of cheap instruction, but rather seek to aid the efficiency and success of the teacher, by allowing him the power, by methodical and inductive modes of instruction, to elevate the standard of professional requirement, and do himself and his subject justice.

If I shall have it in my power to attend the U. S. Association in May (which I fear is not very certain), it will give me great pleasure to be a co-worker in this good enterprise. From the stand point I now occupy of forty years' professional life, I may be permitted to have seen and realized the mischief and tendencies of our lamentably deficient system of public teaching, and sincerely hope that in the good and honest hands into which the subject has now fallen, it may arise renewed, regenerated and disenthralled, and assume a position of public respect and confidence, commensurate with its dignity and importance.

